# Human natural killer cells in major histocompatibility complex class I deficiency

**DOI:** 10.1111/sji.13029

**Published:** 2021-02-21

**Authors:** Neha D. Patil, Maud Theresine, Jacques Zimmer

**Affiliations:** ^1^ CGI Group Department of Infection and Immunity Luxembourg Institute of Health Esch‐sur‐Alzette Luxembourg

We read with interest the personal reflection of. Hans‐Gustaf Ljunggren from the Karolinska Institute (KI) about the path towards natural killer (NK) cell‐based cancer immunotherapy.^1^ The manuscript includes a paragraph about mouse major histocompatibility complex (MHC) class I deficiency, and as we published several papers about the equivalent entity, Human Leukocyte Antigen (HLA) class I defects, we were inspired to briefly remind the NK cell status in these diseases.

Prof. Ljunggren was among the first to describe that NK cells preferentially kill targets with low or absent MHC class I molecules and postulated the existence of MHC class I‐specific inhibitory receptors (IR), which would refrain NK cells from killing normal surrounding cells (missing self‐hypothesis).^2^ The existence of such receptors was demonstrated by several groups in human, rat and mouse.

The researchers from the KI started, among others, to look at mice genetically deficient in MHC class I molecules, such as beta‐2‐microglobulin (β2m) knockout (KO) animals. According to the missing self‐concept, NK cells were expected to kill autologous cells because they lack expression of self MHC class I molecules, but this is not the case at baseline. The NK cells from such animals are tolerant towards autologous targets and unable to perform missing self‐recognition in vitro and in vivo.^3^ These observations were confirmed in transporter associated with antigen processing (TAP) KO mice.^4^

An equivalent to TAP‐deficient mice was described in 1994 by de la Salle et al^5^ in two siblings from a consanguineous marriage, who presented with chronic bacterial infections of the upper and lower respiratory tract. Their serologic HLA class I typing was negative, and the expression of HLA class I molecules assessed by flow cytometry appeared strongly reduced. An autosomal recessive mutation in the TAP‐2 gene was identified. Interestingly, ex vivo NK cells from the patients displayed no cytotoxic activity towards K562 (the classical human HLA class I negative NK cell target) nor towards autologous cells.^5^, ^6^ Thus, these patients’ NK cells were (a) unable to perform missing self‐recognition and (b) tolerant to the autologous MHC class I‐deficient environment. Upon cytokine‐mediated activation, however, they killed several cancer cell lines (including K562), and the autologous B lymphoblastoid cell lines (B‐LCL) and skin fibroblasts.^6^

Moins‐Teissserenc et al (5) and Furukawa et al. (1) described additional TAP‐1 and TAP‐2 deficient patients,^7^, ^8^ of whom some suffered not only from respiratory infections but had also debilitating granulomatous skin lesions or even destruction of the nasal cartilage.^7^ Regarding their NK cells, Furukawa et al confirmed our observations that fresh peripheral blood mononuclear cells were not cytolytic towards K562, Daudi, Molt4 and the HLA class I‐negative B‐LCL 721.221, in contrast to normal effectors.^8^ After activation with interleukin (IL)‐2, IL‐12, or IL‐15, K562 and Molt4 were lysed by the patient's NK cells, but not Daudi nor 721.221, which is opposite to our data.^6^ The authors concluded that the tolerant status of TAP‐deficient NK cells is maintained even after cytokine stimulation to avoid autoreactivity.^8^

Moins‐Teisserenc et al^7^ found that four NK cell clones of one patient were not autoreactive, although they killed K562 targets. In contrast, a NK cell line from another case was autoreactive against B‐LCL, killed the same cell type from another patient but was inhibited by normal B‐LCL, presumably *via* the interaction of HLA class I molecules with specific NK cell IR, the latter being phenotypically and functionally normal in TAP deficiency.^6^

What makes this paper truly interesting and important is the observation that some skin lesions were massively infiltrated with activated NK cells.^7^ This suggests a direct involvement of autoreactive NK cells in the pathogenesis of the lung and skin lesions,^7^ whereas the self‐aggressive peripheral blood NK cells have been stimulated in vitro and were not tested before activation. The initial hypothesis of de la Salle was that the insufficient clearance of viral infections (surprisingly not that severe in TAP‐deficient patients) leads to bacterial colonization and superinfection followed by a chronic and deleterious overactivation of NK cells that cannot be inhibited by the insufficient levels of HLA class I molecules in the environment.

In this context, it is interesting to note that tissue NK cells have become a hot topic in recent years, again partly under the leadership of the KI. It is generally admitted that NK cells in various organs and tissues might not only migrate from peripheral blood, but that different NK cell phenotypes and even lineages might be organ specific.

Although only a bit more than 30 TAP‐deficient patients have been described, the clinical presentation and their NK cells appear quite heterogeneous, as illustrated by the discordant findings of our group and those of Furukawa et al^8^ and Moins‐Teisserenc et al^7^ We encountered a patient with TAP deficiency whose NK cells were cytotoxic ex vivo, who had very severe manifestations and died from cerebral vasculitis. In this case, the dogma of the unlicensed NK cells would not apply and might be explained by the clinical status of the patient.

In addition to TAP deficiency, two cases of human β2 microglobulin deficiency were presented by Ardeniz et al.^9^ In these patients, not only HLA class I expression is reduced, but also that of the CD1a, CD1b and CD1c molecules, of the FcRn receptor and presumably that of the HLA class I‐related molecule MR1, involved in antibacterial defence (as all these structures need to bind β2m for a stable expression). Ex vivo NK cells were not cytotoxic towards K562, in accordance with mouse data (in assays with the appropriate mouse targets).^9^

Overall, NK cells from human HLA class I‐deficient patients seem to behave as their mouse counterparts (hypo‐responsive ex vivo, auto‐aggressive upon cytokine‐mediated activation). In both species, NK cells must be educated by the interaction of IR with their cognate MHC class I ligands to become functional, and, in the absence of this interaction, the cells remain hypo‐responsive. Nevertheless, when they become stimulated in an infectious and inflammatory context, major auto‐aggressive phenomena may occur.

A fundamental difference is that inbred mouse strains are genetically (and maybe even epigenetically) homogeneous, which is not the case when analysing biologic material from different, unrelated human beings. This may explain, at least in part, the discrepancies between our studies and those of Furukawa et al^8^ and Moins‐Teisserenc et al.^7^ In addition, the former stimulated patient cells with cytokines alone for 60 hours,^8^ whereas we applied the method based on the co‐culture of peripheral blood mononuclear cells with irradiated feeder cells (B‐LCL) and IL‐2. Moreover, inbred mice usually live in a pathogen‐free environment, which might account for the absence of a clinical phenotype.

## CONFLICT OF INTERESTS

The authors declare that the present letter was written in the absence of any commercial or financial relationships that could be considered as a potential conflict of interest.

## AUTHOR CONTRIBUTIONS

All authors gave important intellectual input and feedback on the Letter and approved the final submitted version. Jacques Zimmer wrote the Letter.
